# Australian porcine clonal complex 10 (CC10) *Escherichia coli* belong to multiple sublineages of a highly diverse global CC10 phylogeny

**DOI:** 10.1099/mgen.0.000225

**Published:** 2018-10-10

**Authors:** Cameron J. Reid, Matthew Z. DeMaere, Steven P. Djordjevic

**Affiliations:** The i3 institute, University of Technology Sydney, Ultimo, NSW 2007, Australia

**Keywords:** commensal *E. coli*, porcine *E. coli*, clonal complex 10, antimicrobial resistance, microbial genomic epidemiology

## Abstract

We recently identified clonal complex 10 (CC10) *Escherichia coli* as the predominant clonal group in two populations of healthy Australian food-production pigs. CC10 are highly successful, colonizing humans, food-production animals, fresh produce and environmental niches. Furthermore, *E. coli* within CC10 are frequently drug resistant and increasingly reported as human and animal extra-intestinal pathogens. In order to develop a high-resolution global phylogeny and determine the repertoire of antimicrobial-resistance genes, virulence-associated genes and plasmid types within this clonal group, we downloaded 228 publicly available CC10 short-read genome sequences for comparison with 20 porcine CC10 we have previously described. Core genome single nucleotide polymorphism phylogeny revealed a highly diverse global phylogeny consisting of multiple lineages that did not cluster by geography or source of the isolates. Australian porcine strains belonged to several of these divergent lineages, indicative that CC10 is present in these animals due to multiple colonization events. Differences in resistance gene and plasmid carriage between porcine strains and the global collection highlighted the role of lateral gene transfer in the evolution of CC10 strains. Virulence profiles typical of extra-intestinal pathogenic *E. coli* were present in both Australian porcine strains and the broader collection. As both the core phylogeny and accessory gene characteristics appeared unrelated to the geography or source of the isolates, it is likely that the global expansion of CC10 is not a recent event and may be associated with faecal carriage in humans.

## Data Summary

1. All R code used in this study, single nucleotide polymorphism tree files and a fasta database of additional screening genes have been deposited in GitHub (https://github.com/CJREID/CC10_supporting_data).

2. Whole-genome sequenced short reads for the porcine strains in this study are available at Enterobase and the European Nucleotide Archive (ENA); accession numbers are listed in Table S1 (available with the online version of this article).

**Impact Statement**

Clonal complex 10 (CC10) *Escherichia coli* is a commensal gastrointestinal inhabitant in pigs and other food-producing animal species, and is potentially one of the most dominant clonal groups of commensal *E. coli . E. coli* CC10 is found in a wide range of vertebrate hosts as a commensal in the gastrointestinal tract and in aquatic environments, and carries diverse antimicrobial-resistance (AMR) genes and is known to cause extra-intestinal infections; however, the global epidemiology of this lineage has yet to be explored. Here, we provide a preliminary snapshot of the global phylogeny, AMR, virulence and plasmid gene carriage of CC10 *E. coli *. The work provides a reference point for further study of CC10 *E. coli* and illustrates that multiple lineages are globally disseminated. Due to its global range, intestinal carriage in both humans and animals, and wide variety of AMR and virulence genotypes, CC10 *E. coli* should be closely monitored in the context of public health.

## Introduction

*Escherichia coli* is both a successful commensal and a serious pathogen affecting human and animal health, and is the most frequently isolated Gram-negative pathogen impacting human health [1]. Multidrug-resistant infections (resistant to three or more classes of antimicrobials) are forecast to cause 10 million deaths per year by 2050, and it is expected that *E. coli* will be responsible for 30 % of fatalities and 40 % of projected economic losses that arise as a consequence [2]. Pathogenic *E. coli* may infect intestinal sites (InPEC; intestinal pathogenic *E. coli *) or extra-intestinal sites (ExPEC; extra-intestinal pathogenic *E. coli *) [3] and antimicrobial resistance (AMR) often complicates treatment, increasing rates of morbidity and mortality [4]. Severe outbreaks of drug-resistant InPEC disease, such as the O104 : H4 outbreak in 2011, are well documented, as well as the global dissemination of resistant ExPEC clones, such as ST131 [5–7]. Currently, responses to such events are reactive, and come after significant financial and human cost is incurred. It would, therefore, be beneficial to be able to identify, monitor and track populations of *E. coli* that pose a threat to human health, so that risks can be predicted and strategies implemented to mitigate the impact. To this end, the emerging field of genomic epidemiology is critical, as it allows the highest resolution of microbial population structure and genetic determinants of virulence and AMR. By developing global databases of genomic, phenotypic, spatial and temporal information for *E. coli ,* predictive disease management is a distinct possibility for the near future [8, 9].

There is currently much debate about the contribution of different reservoirs of *E. coli* to disease and AMR carriage in humans [9–13]. Food-production animals and associated retail meats have been widely investigated and genetic similarities documented; however, the data is often limited to multilocus sequence typing (MLST) types and PCR identification of a select number of genes [14, 15]. Furthermore, the sample sizes used are a limiting factor in the significance of the conclusions that may be reached. Nevertheless, these studies provide a good starting point for further genomic investigation.

We recently described the phylogeny, virulence-associated gene (VAG) and AMR gene (ARG) carriage in a collection of 103 *E. coli* genome sequences derived from the faeces of healthy Australian pigs [16]. This study identified clonal complex 10 (CC10) as the predominant lineage within the collection. These CC10 strains were phylogenetically diverse and carried multiple ARGs and VAGs associated with ExPEC. CC10 is globally reported as a resident of the intestinal tract of humans, food-production animals, companion animals and wild animals [14, 17]. It has also been identified in retail meats and plant-based foods, as well as wastewater, rivers and urban streams [18, 19]. Furthermore, CC10 can cause extra-intestinal disease in pigs, dogs and humans [14, 17, 20]. AMR, including extended-spectrum β-lactamase (ESBL) carriage, is also widely reported [19, 21]. These observations suggest a clonal complex with broad fitness characteristics, a wide host range, pathogenic potential and a variety of AMR traits. Despite the wealth of literature reporting CC10, the global population structure and diversity of VAGs and ARGs remains unknown.

The aim of this study was firstly to examine a global collection of CC10 *E. coli* genome sequences to determine the population structure and the diversity of VAGs, ARGs and plasmid replicons. Secondly, we aimed to determine how Australian porcine CC10 *E. coli* relate to the global phylogeny, and to compare VAG, ARG and plasmid replicon carriage to the existing global collection.

## Methods

### Genome sequences used in this study

The 20 Australian porcine faecal-derived *E. coli* CC10 strains included in this analysis were whole-genome sequenced using a modified Nextera protocol and the Illumina HiSeq platform, as previously described [16]. These strains retain their original names from the previous publication and are preceded by ‘F2_’. The 20 strains described in this paper are available as short reads in Enterobase (http://enterobase.warwick.ac.uk) and the European Nucleotide Archive. All accession numbers are listed in Table S1.

Publicly available *E. coli* CC10 Illumina short reads were downloaded from Enterobase (accessed 22/11/17). The database was queried for CC10 and a summary spreadsheet was downloaded in order to select sequences from well-defined sources, with the desired metadata for which full short reads were available. Accepted sources were: (i) animal including faeces (pig, cattle, poultry, horse, dog); (ii) food (pig, cattle, poultry, dairy cheese, plant); (iii) human including faeces, urine and blood; and (iv) environment including soil and wastewater. Furthermore, metadata was required for the year of isolation, and country and continent of origin. Sequences derived from laboratory strains were excluded, as were sequences with ambiguous or contradictory source details. This spreadsheet was then used to query the National Center for Biotechnology Information sra and download read sets using the download_enterobase_SRA_reads.sh script available at https://github.com/bogemad/snp_phylogeny. Enterobase sequences were named for analysis by removing the prefix ‘ESC_’ and suffix ‘AA’ from their ‘Uberstrain’ accession numbers (Table S1). Preliminary analysis indicated a number of clones were present in the collection; therefore, we selected a single representative strain in cases where groups of sequences were separated by ≤3 single nucleotide polymorphisms (SNPs), and had identical plasmid replicon and resistance-gene profiles. The final collection numbered 248 sequences, comprising 228 from Enterobase and 20 from our previous study.

### Phylogenetic analysis

Snippy v4.0.2 (https://github.com/tseemann/snippy) was used with default parameters to map short reads from the 248 CC10 strains, as well as phylogroup A non-ST10 strain HS (gb|CP000802.1) to ST10 reference sequence K12-MG1655 (gb|U00096.3). A core genome alignment was then generated using the snippy-core function. This function generates two alignments, a ‘full core’ alignment of all reads to the reference genome and a ‘core SNP’ alignment consisting only of SNP sites present in all genomes, ignoring insertion and deletion variant types. Both of these alignments were used to generate maximum-likelihood trees. Files available as supporting data (see Data Summary) are hereafter named in parentheses. The full alignment was cleaned using the snippy-clean_full_aln function and filtered for recombination using default settings with Gubbins v2.3.4 [22] resulting in an alignment of 79 039 sites (full.core.clean.gubbins.aln). Variable positions present in all strains were then identified with SNP-sites v2.4.0 [23]. The final alignment consisted of 4515 SNP positions (full.core.clean.gubbins.snpsites.aln). The core SNP alignment generated by snippy (snippycore.aln) consisted of 72 136 sites. FastTree2 v2.1.10 [24] was used to generate maximum-likelihood phylogenetic trees under a GTR (generalised time-reversible)nucleotide substitution model with default settings for both of these alignments (full.core.clean.gubbins.snpsites.tree and snippycore.tree). The trees were visualized with metadata in iTOL 4.2.3 [25]. The full core tree was also rooted in FigTree v1.4.2 (http://tree.bio.ed.ac.uk/software/figtree/) and then visualized alongside gene-screening heatmaps in R (version 3.3.1) with ggtree 3.6 (full.core.clean.gubbins.snpsites.rooted.tree) [26]. Pairwise SNPs between all strains were extracted from the recombination filtered full core alignment using the snp_phylo_utils script available at https://github.com/bogemad/snp_phylogeny. All trees, alignments used to generate them, summary statistics from snippy-core and the pairwise SNP table are available at https://github.com/CJREID/CC10_supporting_data.

### Gene screening

ARGs, VAGs, plasmid replicon genes and OH antigen genes were identified using ariba (version 2.10.1) [27] with ResFinder, PlasmidFinder, VirulenceFinder and SerotypeFinder databases available from the Center for Genomic Epidemiology (http://www.genomicepidemiology.org/) [see DataBibliography]. Pasteur MLST types were also determined with ariba’s built-in MLST typing function. An additional custom database of further virulence- and resistance-associated genes not present in the aforementioned databases was also used. This database is available at https://github.com/CJREID/CC10_supporting_data. Gene presence or absence was then visualized with the phylogenetic tree in ggtree [26].

### Multidimensional scaling (MDS) analysis

In order to determine whether virulence or resistance and plasmid gene carriage was related to continent of isolation, sequence type or origin of CC10 strains, we conducted a non-metric MDS analysis in R using mass and vegan packages. Briefly, the Jaccard index was used to produce a pairwise distance matrix for absence/presence profiles of virulence and combined resistance and plasmid replicon genes for each strain. MDS was performed using the methods mass::isoMDS [28] in combination with vegan ::initMDS and vegan :: postMDS [29], with default settings. First, metric scaling was assumed to generate a baseline solution in two dimensions before iterating the same process with initMDS to reduce the stress value. postMDS was then used to standardize the final configurations for ease of interpretation. ggplot2 was used to plot the ordinations with clusters keyed separately on origin, sequence type and continent of isolation. A normal distribution was assumed to infer 95 % confidence interval (CI) ellipses. All R code used in this study is available at https://github.com/CJREID/CC10_supporting_data.

### R package versions

The R package versions used were ape 5.0, mass 7.3, vegan 2.4-5, dplyr 0.7.1, reshape2 1.4.2, grid 3.3.1, ggtree 1.6.11, ggplot2 2.2.1.

## Results and Discussion

**Fig. 1.**
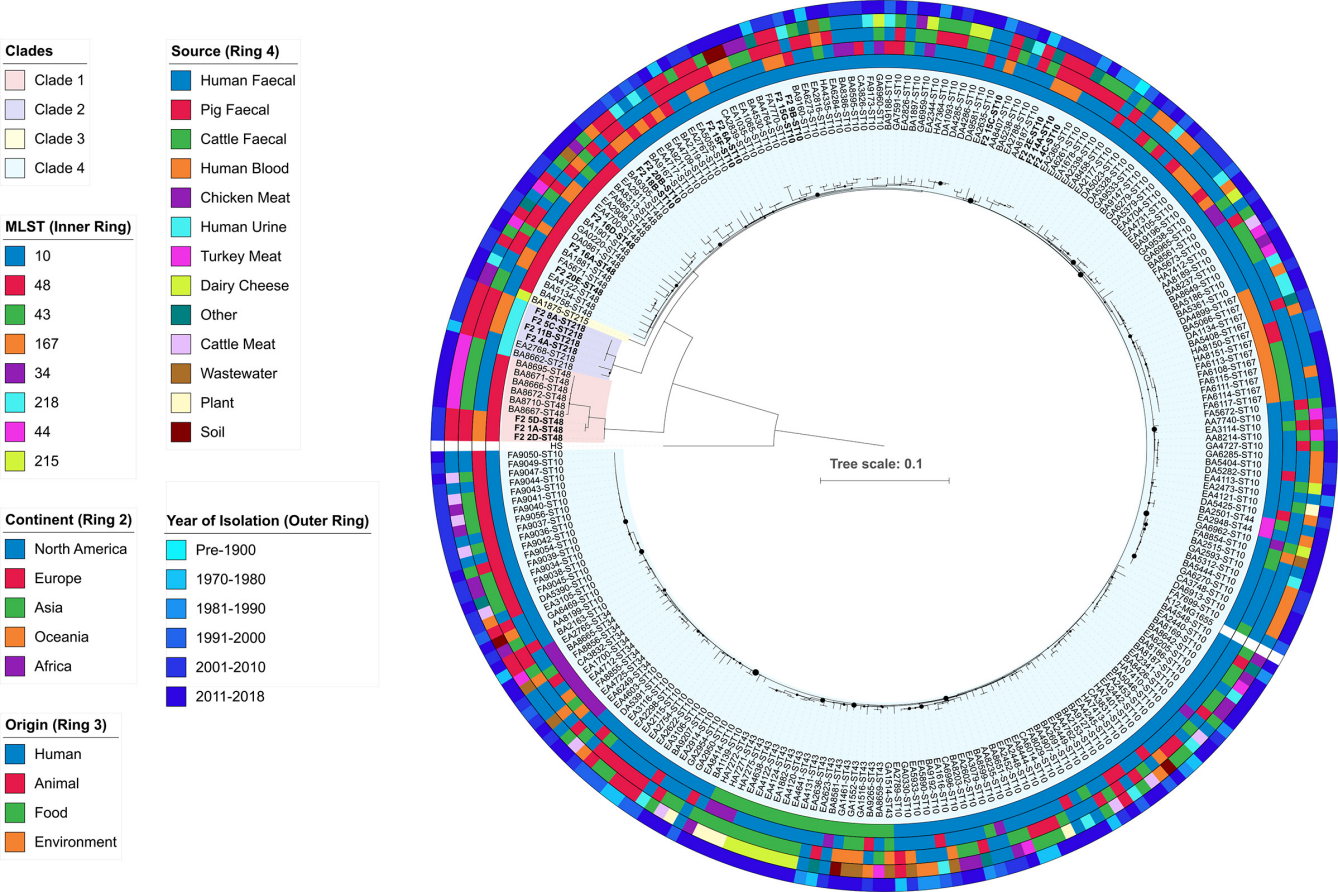
Maximum-likelihood phylogeny of 248 *E. coli* CC10 sequences. Australian porcine sequences are in bold. Small nodes indicate high confidence splits, whilst larger nodes indicate lower confidence splits. Sequence types, continent of isolation, origin of isolates, source of isolates and year of isolation are annotated on the coloured outer rings according to the key. Clades are also coloured according to the key. Metadata for reference strain K12-MG1655 and outgroup strain HS is omitted.

The collection of CC10 sequences (*n*=248) encompassed eight sequence types (Achtman) from five continents. Pasteur sequence types were also determined; however, concordance between the two schemes was highly variable (Table S1). Sequences were classified as being of food, animal, environment or human origin (Table 1). Strains were further classified into 16 sources, though 4 of these, for which there were less than 10 representatives, were grouped as ‘other’ for analysis (Fig. 1, Table S1). The collection was heavily weighted towards North American samples; however, the distribution between animal, food and human sources was fairly even. Environmental samples were scarce. Isolation dates mostly ranged from 1979 to 2017, though one sequence was derived from a strain isolated in 1895. A total of 122 serotypes were predicted and 10 of these were O-non-typeable (Table S1).

**Table 1.** CC10 sequences used in this study

Source	Sequence type								Total
	10	34	43	44	48	167	215	218	
Africa									13
Food	4		3						
Human	5		1						
Asia									23
Animal	2								
Environment	2	2	3		1				
Human	6			1		6			
Europe									46
Animal	5								
Food	10								
Human	27	1	1			2			
North America									136
Animal	37	2	1		4	1		1	
Environment	6		1						
Food	33	1	8		10		1	1	
Human	21	1	1	1	2	3			
Oceania									30
Animal	10				6			4	
Human	6	2			2				
Total source									248
Animal	54	2	1		10	1		5	73
Environment	8	2	4		1				15
Food	47	1	11		10		1	1	71
Human	65	4	3	2	4	11			89
Total sequence type	174	9	19	2	25	12	1	6	248

### Global phylogeny of *E. coli* CC10

In order to understand the population structure of our collection of CC10 isolates, a whole-genome alignment core SNP phylogeny was generated using the completed genome of ST10 *E. coli* K12-MG1655 as a reference and the complete genome of ST46 phylogroup A *E. coli* HS as an out-group strain. The mean number of bases aligned to the reference in the full core alignment was 4 272 312/4 643 559 (92 %). Recombination filtering reduced this alignment to 79 039 bases and SNP identification resulted in a final alignment of 4515 variable sites present in all strains. A maximum-likelihood tree was built from this alignment and the tree comprised four well-supported major clades. Clade 1 comprised only ST48 strains. This clade was separated from clade 2 by approximately 450 core SNPs. Interestingly, another cluster of ST48 strains was present in clade 4, differing from ST10 strains by approximately 90 core SNPs. This suggests that ST48 comprises two separate evolutionary lineages that may have been separated by geographical or host-isolation for some time before disseminating again. A maximum-likelihood tree generated with the snippy-core core SNP alignment also supported this topology, albeit with some rearrangements in clade 4 (Fig. S1). This high-resolution approach supports previous work on these strains, in which Phylosift analysis also separated ST48 strains in the context of a wider collection of sequence types [27]. We are unaware of any other phylogenomic studies that demonstrate such a split between members of the same sequence type. However, intra-sequence type diversity, divergence and instances where core genome alignment does not strictly follow preceding typing schemes have been reported [7, 30, 31]. It is difficult to compare results between studies due to different methodologies, the effect of different reference genomes and the number of strains included in the analysis. Nonetheless, this result supports whole-genome core SNP phylogeny together with MLST to accurately resolve clonal groups [30].

ST218 strains formed the second clade, whilst a single ST215 formed a third clade. Clade 4 was the largest and most diverse with respect to sequence types, containing ST10, ST34, ST43, ST44, ST48 and ST167. Major splits in the phylogeny were well supported; however, some poor node confidence values were present within the sub-clades of clade 4, usually at splits between ST10 strains. The major lineages did not cluster sequences based on continent of isolation, origin or source (Fig. 1). There were numerous examples of sequences from disparate sources, origins and continents being closely related. This data supports a diverse clonal group consisting of multiple lineages with broad fitness characteristics that is globally dispersed and capable of inhabiting a wide variety of niches.

### Australian porcine *E. coli* CC10 sequences in the context of global phylogeny

Thirteen Australian porcine sequences belonged to clade 4 (*n*=13), while the remaining seven ST48 and ST218 sequences belonged to clades 1 and 2, respectively. This indicates that CC10 lineages have been introduced to Australian pigs multiple times. Within clade 4, a number of Australian porcine sequences clustered with temporally and geographically unrelated sequences. Sequences that clustered with Australian porcine strains included human blood, urine and faecal isolates, and turkey meat, chicken faecal and chicken meat isolates. This observation supports a growing body of literature that suggests CC10 *E. coli* are capable of colonizing a broad range of hosts in both commensal and pathogenic capacities, and does not preclude the possibility that porcine-origin CC10 are capable of causing extra-intestinal infection [14, 32].

Interestingly, Australian porcine sequences were mostly present on different sub-clades to the 14 other porcine faecal strains in the collection, indicating divergence and diversity among CC10 strains that colonize pigs. However, two examples of closely related pig-derived strains were present in clade 4. Porcine faecal strain EA2788, isolated in 1979 in the USA, was separated by 25 core SNPs from its closest Australian relative F2_2E. Strain AA8187, isolated in the USA in 2009 from pig meat was yet more closely related to F2_2E, separated by only nine core SNPs.

These three sequences possessed similar VAG profiles; however, their resistance-gene profiles were vastly different. The Australian strain carried 15 ARGs compared to 7 in AA8187 and 3 in EA2788. Furthermore, F2_2E carried a full suite of IncF replicons (FII, FIA, FIB, FIC), whilst AA8187 carried an IncFIA and an IncHI1 replicon, and EA2788 carried an IncY replicon. These observations indicate that some CC10 strains are capable of persisting for long periods of time in association with the porcine gut. Whilst the actual genetic determinants of fitness in the porcine gut are not known, the phylogenetic similarity between these three strains likely reflects carriage of similar, if not identical, fitness factors. This persistence has previously been described between sows and their piglets, and is likely to persist on a global scale due to the common ancestry of all domestic pig breeds [33, 34]. It would be interesting to see whether the similarity between these Australian and American porcine strains is reflected in CC10 isolates from China, which are underrepresented in this collection. China is the world’s largest producer of pork [35] and transfer of similar strains is likely to have occurred through trade of meat products and human travel between the two countries. A broader, globally sourced collection of porcine CC10 sequences would be useful to characterize dominant strains and determine the underlying genetic basis of their fitness. In contrast to the conservation of closely related strains, it is evident among pig-associated strains that variability still arises in the form of accessory gene content such as plasmids and ARGs, likely due to differential antimicrobial selective pressures between pork production systems worldwide.

### CC10 strains carry a wide variety of VAGs

**Fig. 2.**
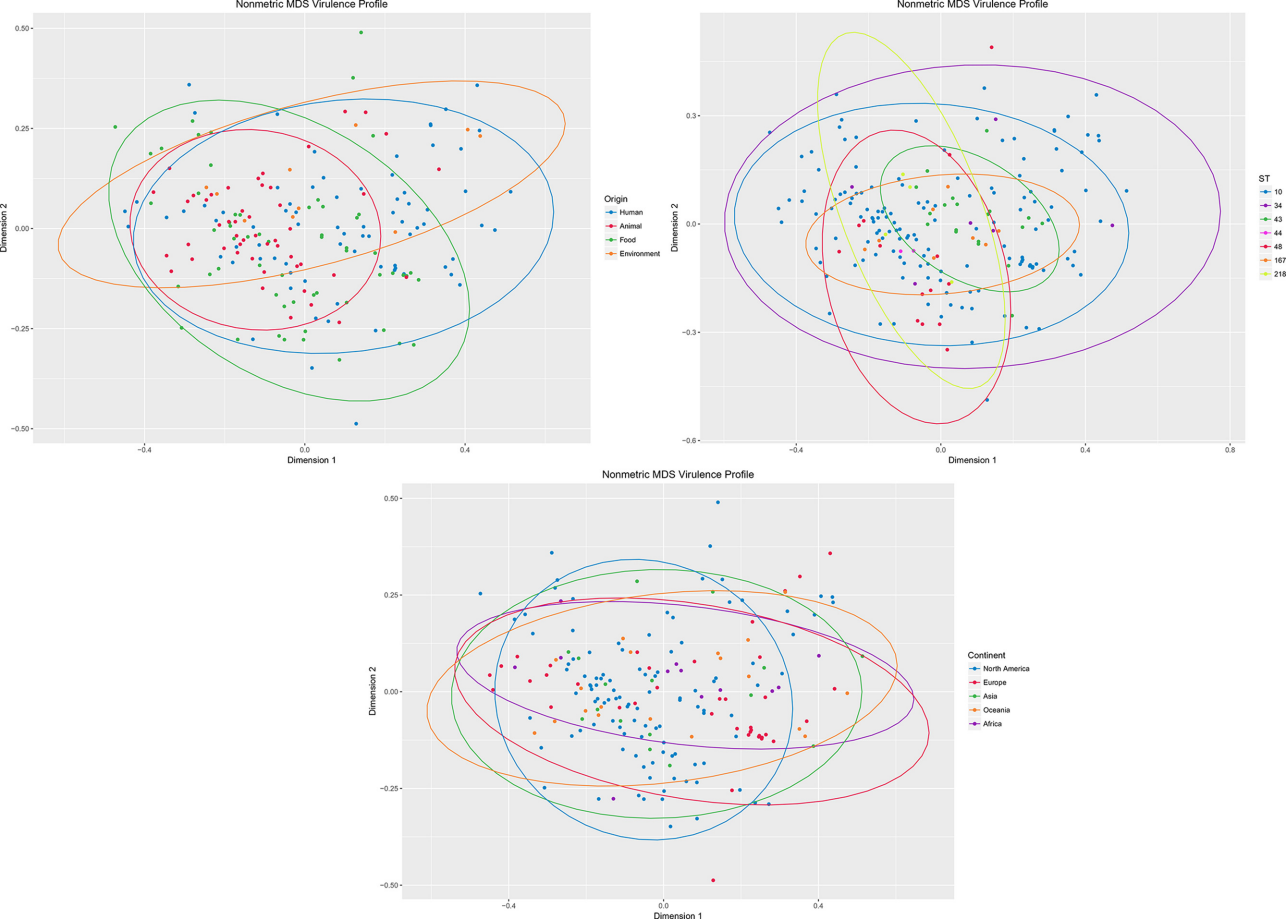
Non-metric MDS graph of 194 unique virulence gene profiles grouped by 95 % CI ellipses for origin of the isolate, sequence type and continent of isolation. NB Some characteristics contained too few data points for ellipses to be calculated.

We identified a total of 110 VAGs within the collection. Strains carried between 2 and 34 VAGs, with a mean of 10 VAGs per strain, revealing a wide variety of VAG profiles. The mean number of VAGs per strain by source was highest in humans (*n*=12), followed by food and environmental (*n*=10) and animals (*n*=7). No clear relationship between the VAG profiles and source or geography was evident when gene presence/absence was mapped alongside the SNP phylogeny (Fig. S2). Some closely related strains exhibited similar virulence profiles; however, they were rarely identical. We therefore conducted a non-metric MDS analysis of 194 unique virulence profiles by continent of isolation, sequence type and origin. This analysis did not separate strains based on any of these characteristics, suggesting that virulence profiles are detached from these factors (Figs 2 and S2) It should be noted that small sample sizes for some groups may obscure the true distributions inferred by the ellipses; however, this was unable to be avoided due to the limitations of publicly available sequence data. Overall, these observations highlight significant diversity in the virulence potential of CC10 strains and suggest lateral gene transfer and homologous recombination events that are unrelated to core phylogeny or source of isolation play a major role in CC10 diversity [36]. This also indicates that the underlying genetic factors responsible for the fitness and global spread of CC10 are unlikely to be related to our current understanding of virulence potential. This is an area for further investigation.

ExPEC-associated genes were among the most common genes detected in the collection. These included *fimH* (*n*=206, 83 %)*, traT* (*n*=148, 60 %)*, iss* (*n*=134, 54 %) and iron-acquisition-associated genes such as *irp2, fyuA, sitA, iutA* and *iucD* (*n*=88, 35 %; *n*=92, 37 %; *n*=56, 23 %; *n*=23, 9 %; *n*=44, 18 %; respectively)*. pap* operon pilus-assembly and *kpsMTII* capsular antigen genes were also detected. Carriage of ExPEC virulence factors has been previously reported in CC10 and may reflect the association of many of these genes with intestinal fitness [37], conversely it may imply that innately fit and widespread lineages are simply more likely to acquire virulence genes. VAGs associated with enteric pathotypes were uncommon by comparison to ExPEC factors. Only 14 strains carried an *stx* variant characteristic of Shiga-toxigenic *E. coli* (STEC). One of these carried both *stx1B* and *stx2B.* Subtypes were not determined. These genes are typically acquired via bacteriophage transduction [38]. This observation highlights that abundant clonal groups such as CC10 may acquire virulence traits typical of both ExPEC and InPEC.

The mean number of VAGs (*n*=6) in Australian porcine faecal strains was lower than the collection mean (*n*=10). The most common ExPEC VAGs among porcine faecal strains were *fimH* (*n*=16/20, 80 %), *traT* (*n*=15/20, 75 %) *hek, irp2* and *fyuA* (all *n*=5/20, 25 %). VAGs of intestinal pathotypes were not identified. Overall, the low abundance of VAGs suggests low virulence potential of these strains. In contrast, CC10 strains have been isolated from pigs with extra-intestinal infections in China, suggesting that porcine CC10 may acquire VAGs that cause ExPEC in the same host [32]. It is unknown whether these strains have developed virulence in the porcine gut via lateral gene transfer or whether they are virulent strains introduced by humans to porcine production environments.

### Resistance genes

We identified 73 ARGs in the total collection, with a range of 0 to 19 ARGs per strain and a mean of 5 per strain (Table S1). The mean number of ARGs by origin was 7 for environmental, 6 for animals and humans, and 4 for food-derived strains. Class 1 integrase gene *intI1* was present in 77 strains (31 %), whilst class 2 integrase *intI2* was present in 29 strains (12 %). Genes conferring resistance to older classes of antibiotics, such as streptomycin (*strB/aph(*6′*)-Id*, *n*=105, 42 %; *strA/aph*(3′*)-Ib*, *n*=113, 46 %), penicillin (*bla*_TEM-1B_; *n*=93, 38 %), aminoglycosides (*aadA1*; *n*=65, 26 %), tetracycline (*tetA*; *n*=105, 42 %) and sulphonamides (*sul2*; *n*=98, 40 %), were most common in the collection. ESBL genes were not common; however, they were represented by a variety of genes, notably *bla*_CTX-M-32_ (*n*=17, 7 %), *bla*_CTX-M-15_ (*n*=11, 4 %), *bla*_CMY_ (*n*=13, 5 %) and *bla*_OXA1_ (*n*=10, 4 %).

ESBL carriage in this collection of CC10 is diverse, but not particularly abundant. This is interesting given numerous reports in the literature pointing to CC10 as a common source of ESBL genes [21, 39, 40]. This once again demonstrates the current limitations of data-mining and the need for more publicly available sequences to develop an accurate understanding of ESBL carriage in CC10 and *E. coli* collectively. This is particularly illustrated by the fact that ST10 was identified as the most common ESBL-positive sequence type in Taiwanese river water [18]. The ability of CC10 to survive in river water has implications for its ability to disseminate, and increases its exposure to diverse niches and ability to spread ESBL genes.

Colistin-resistance gene *mcr1* was present in five sequences, four of which originated from wastewater, a known reservoir of ARGs and mobile elements that transfer them [41]. Like ESBL carriage, *mcr* carriage in CC10 is commonly reported in the literature in contrast to the collection examined here [42–44]. A 2018 study by García and colleagues found that ST10 was the primary carrier of *mcr-4* and *mcr-5* variants in a collection of colistin-resistant porcine enterotoxigenic *E. coli* (ETEC) in Spain. This highlights the danger of exposing a successful lineage like CC10, within which the line between commensalism and pathogenicity is ill defined, to critically important antibiotics, as they are likely to acquire resistance rapidly.

The mean number of ARGs for Australian porcine CC10 was 11, more than double that of the collection as a whole. This discrepancy is likely a result of the extensive history of antimicrobial use at the farm where the strains were isolated and selection of *intI1*-positive strains as a proxy for multiple-drug resistance [45]. A family of related integrons was abundant in this collection and responsible for a proportion of the extensive resistance [16]. The carriage of *sul3* in the Australian porcine strains was high (*n*=13/20; 65 %) relative to the rest of the collection (*n*=11/228; 5 %). This is likely a reflection of the lower carriage of class 1 integrons in the overall collection compared to the Australian isolates included in this study (all *intI1* ^+^) as *sul3* is associated with atypical class 1 integrons [16]. Carriage of *sul3* has an established association with *E. coli* from porcine sources [46]. ESBL and *mcr* carriage was not observed in the Australian strains. It is difficult to compare our Australian porcine strains to the global strains with respect to AMR; however, it is clear that AMR depends on the exposure history of the strain as opposed to its physical or geographical source.

### Plasmid replicons

We identified 21 plasmid Inc replicons in the collection. Strains carried between zero and seven replicons with a mean of two. IncF replicons dominated, FII (*n*=163, 66 %), FIB (*n*=122, 49 %), followed by IncI1 (*n*=66, 27 %) and IncX1 (*n*=45, 18 %). IncF and IncI1 plasmid families in particular are frequently implicated in the spread of ARGs and are likely to play a role in the observed ARG carriage in this collection of CC10 [47]. The global expansion of ST131 ExPEC provides a cautionary tale in underestimating the potential for faecal commensal strains to pose a threat to human health. Like most ExPEC, ST131 is associated with human faeces and multiple IncF plasmids that carry extensive arrays of drug-resistance genes. ST131, in conjunction with these plasmids, has expanded to become the predominant ESBL-producing clone in hospital- and community-acquired ExPEC infections [7, 48]. It is conceivable that such a plasmid expansion event associated with a CC10 strain could yield a conjugative plasmid with serious AMR and virulence characteristics. As CC10 is a common faecal commensal of both animal and humans, can cause human ExPEC infections, inhabit diverse environmental niches and carry a wide variety of resistance-associated plasmids, it is critical that this lineage is monitored in the context of human health.

Australian porcine CC10 also carried IncF replicons: FII (*n*=13, 65 %), FIB (*n*=13, 65 %), IncI1 (*n*=7, 35 %) and IncX1 (*n*=10, 50 %) replicons. It is not known whether these plasmid types have been acquired on multiple occasions by CC10 or have remained stable in sub-lineages over long periods of time. IncHI2 was notably carried by eight strains (40 %), and was abundant in the larger collection of previously described porcine strains from Australia [16]. This is in contrast to the rest of the CC10 collection, where IncHI2 was only present in 11 other strains. The IncHI2 replicons in the Australian porcine strains were all of the same pMLST (plasmid-based multilocus sequence typing) type ST3, suggesting a localized plasmid-acquisition event. This is supported by reports of highly related IncHI2 plasmids with multiple resistance genes circulating in both *E. coli* and *Salmonella* sp. in the Asia Pacific region [49, 50]. This once again highlights the ability of CC10 and successful commensals to acquire diverse plasmids that carry drug-resistance genes.

**Fig. 3.**
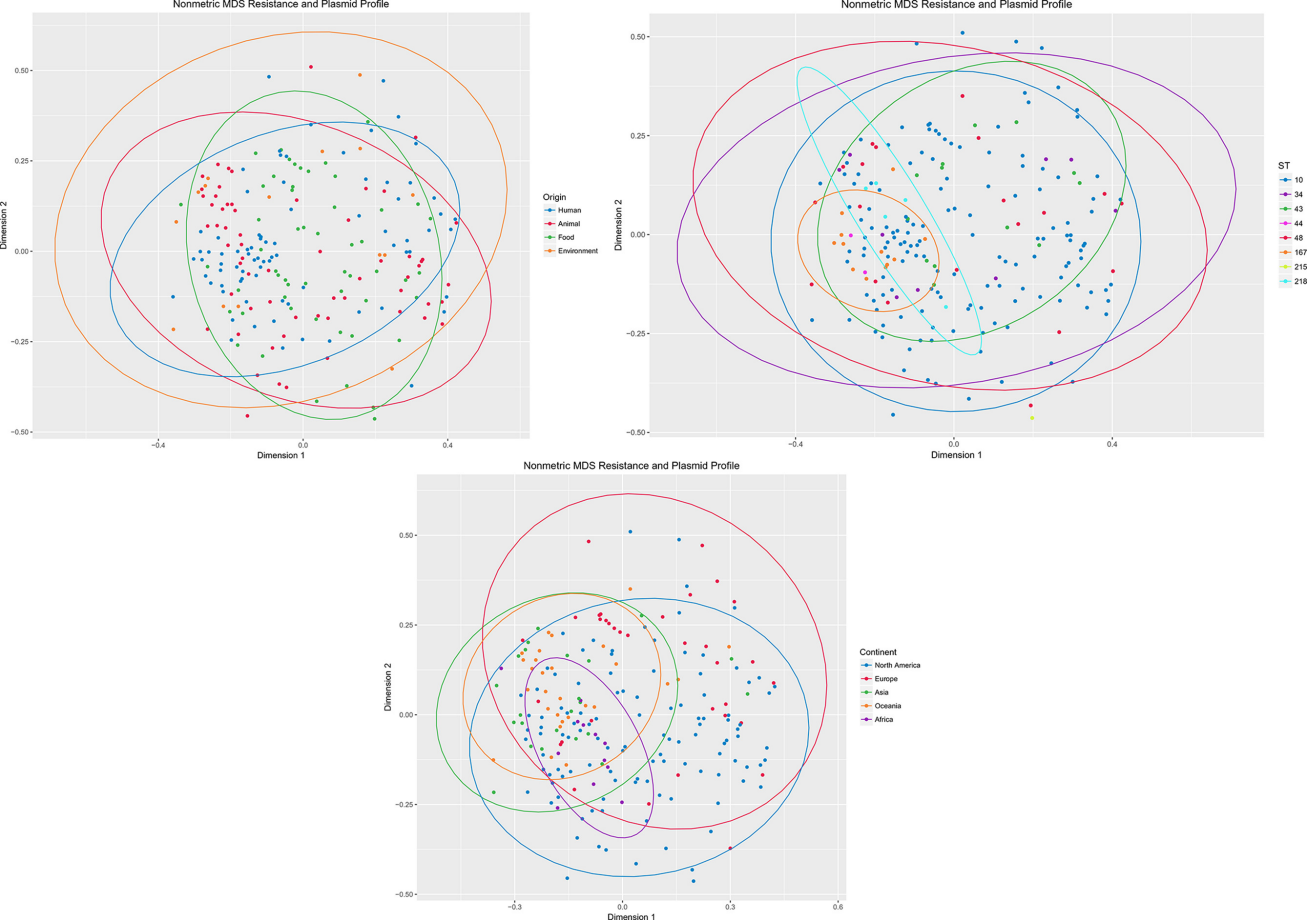
Non-metric MDS graph of 211 unique plasmid replicon and ARGs profiles grouped by 95 % CI ellipses for origin of the isolate, sequence type and continent of isolation. NB Some characteristics contained too few data points for ellipses to be calculated.

Mapping of plasmid and resistance-gene carriage to the SNP tree did not appear to link phylogeny or origin with gene carriage (Fig. S3). Furthermore, MDS analysis of 211 unique plasmid replicon and resistance-gene profiles did not cluster strains based on continent of isolation, sequence type or origin (Fig. 3). Similar to the case with virulence genes, it appears that due to the widespread and diverse nature of CC10, accessory-gene carriage is not associated with the geography or source of the isolates. However, it is possible that trends could be observed if sample geography or source was restricted. Similarly, trends might emerge if a larger, more comprehensively sampled collection were analysed. Further studies with controlled sampling and sufficient metadata are required to explore this.

### Conclusions

This genomic analysis has demonstrated that CC10 is highly diverse with respect to the core genome as well as accessory elements. This diversity is illustrated by its host and geographical range and suggests a core set of fitness traits that are yet to be genetically defined and characterized. It is likely, as in the case of other global lineages of *E. coli *, that recombination and genomic islands in the chromosome play a role in its success. These attributes should be investigated in future work. Whilst this study is relatively large in scale, it is limited by our inability to control and balance sample sizes from different sources and geographical regions. As online databases of whole-genome data continue to grow, the scale of these studies should be expanded to elucidate epidemiological features that cannot be determined currently. We suspect the apparent abundance of CC10 in human faeces indicates humans are the predominant intermediaries between strains found in other animals and environments. The dispersion of Australian porcine sequences throughout the phylogeny indicates they are derived from multiple lineages within CC10 that are well adapted to the porcine gut. The variety of VAGs and ARGs suggests that these mobilized genetic traits are decoupled from phylogeny and depend instead on the history of each individual strain.

## Data bibliography

1. Zankari E, Hasman H, Cosentino S, Vestergaard M, Rasmussen S *et al*. Identification of acquired antimicrobial resistance genes. *J Antimicrob Chemother* 2012;67:2640–2644.
2. Joensen KG, Scheutz F, Lund O, Hasman H, Kaas RS *et al*. Real-time whole-genome sequencing for routine typing, surveillance, and outbreak detection of verotoxigenic *Escherichia coli *. *J Clin Microbiol* 2014;52:1501–1510.
3. Carattoli A, Zankari E, Garcia-Fernandez A, Voldby Larsen M, Lund O *et al*. *In silico* detection and typing of plasmids using PlasmidFinder and plasmid multilocus sequence typing. *Antimicrob Agents Chemother* 2014;58:3895–3903.
4. Joensen KG, Tetzschner AM, Iguchi A, Aarestrup FM, Scheutz F. Rapid and easy in silico serotyping of *Escherichia coli* isolates by use of whole-genome sequencing data. *J Clin Microbiol* 2015;53:2410–2426.

## Supplementary Data

Supplementary File 1

Supplementary File 2
