# Supplementary material for: Australian porcine clonal complex 10 (CC10) Escherichia coli belong to multiple sublineages of a highly diverse global CC10 phylogeny
Source: Microb Genom. 2018 Oct 10;5(3):e000225. doi: 10.1099/mgen.0.000225 (PMC6487311; doi:10.1099/mgen.0.000225)
Supplement: Supplementary File 1 [file mgen-5-225-s001.pdf]

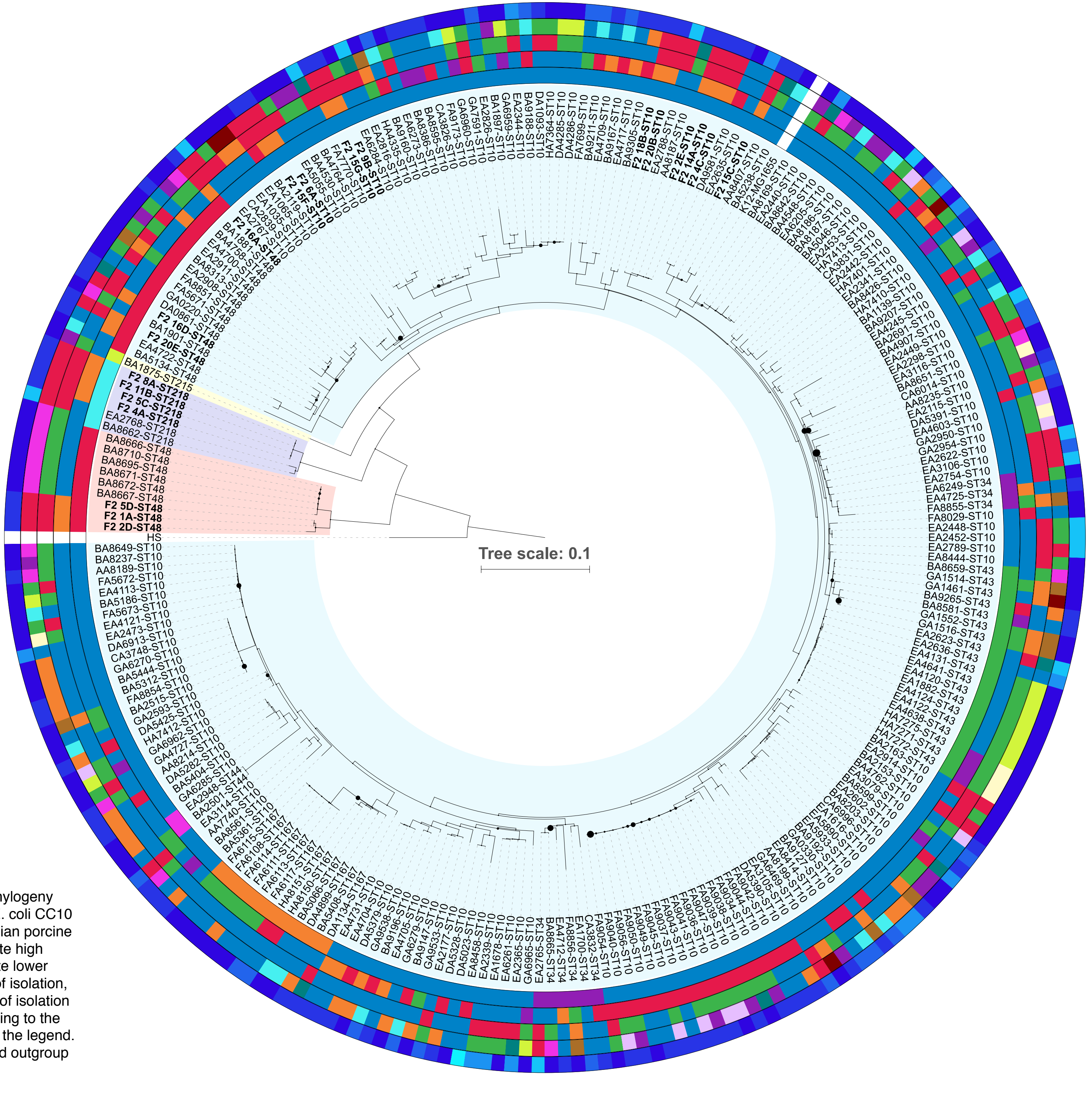

Figure S1. Maximum-likelihood core SNP phylogeny generated from snippy-core alignment of 248 E. coli CC10 sequences (branch lengths not shown). Australian porcine sequences are in bold. Small nodes indicate high confidence splits whilst larger nodes indicate lower confidence splits. Sequence types, continent of isolation, origin of isolation, source of isolation and year of isolation are annotated on coloured outer rings according to the legends. Clades are also coloured according to the legend. Metadata for reference strain K12-MG1655 and outgroup strain HS is omitted.

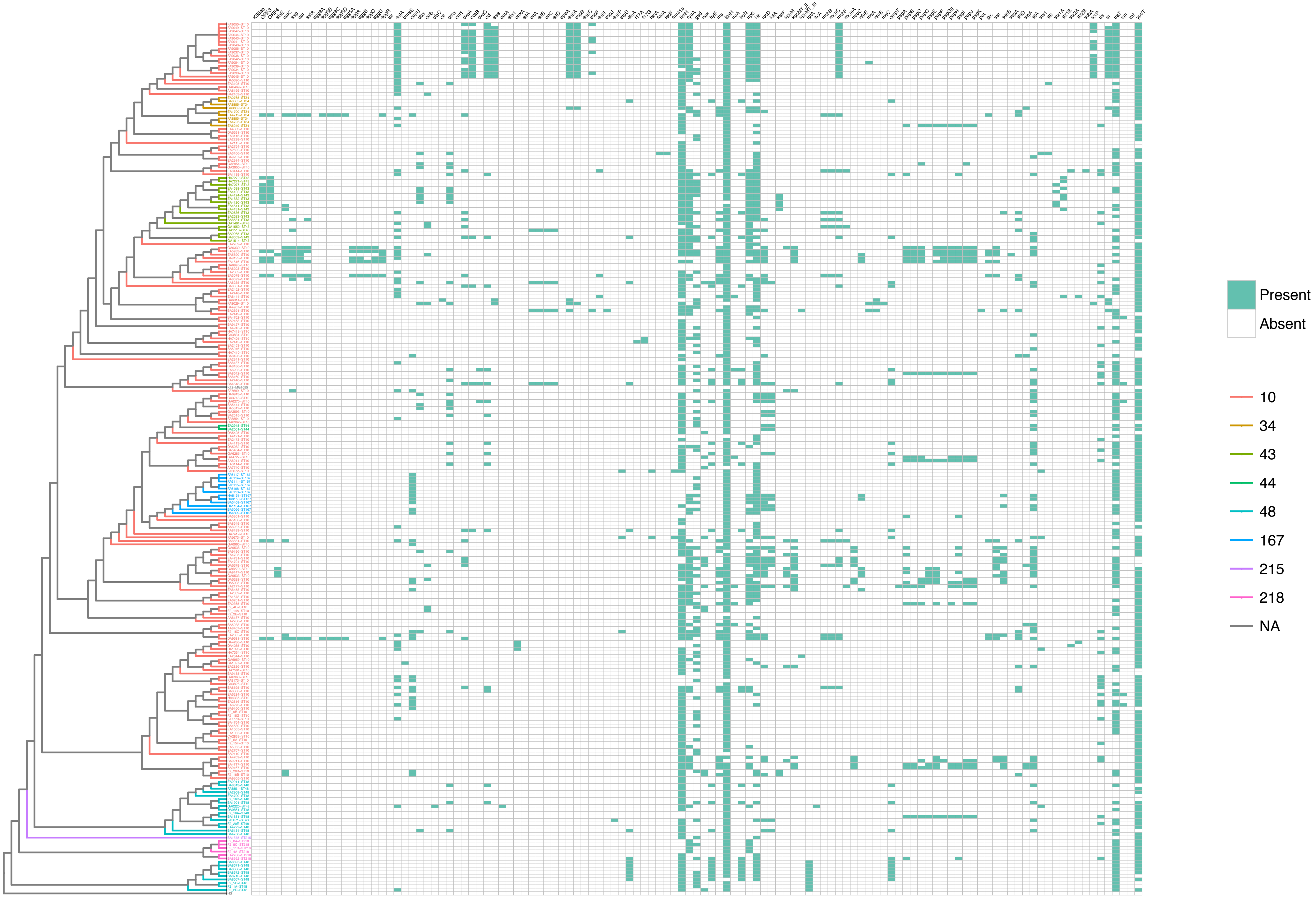

Figure S2. Heatmap depicting maximum-likelihood phylogeny from recombination filtered full core alignment (shown in figure 1) mapped against virulence gene presence and absence. Tips are coloured by Achtman sequence type.

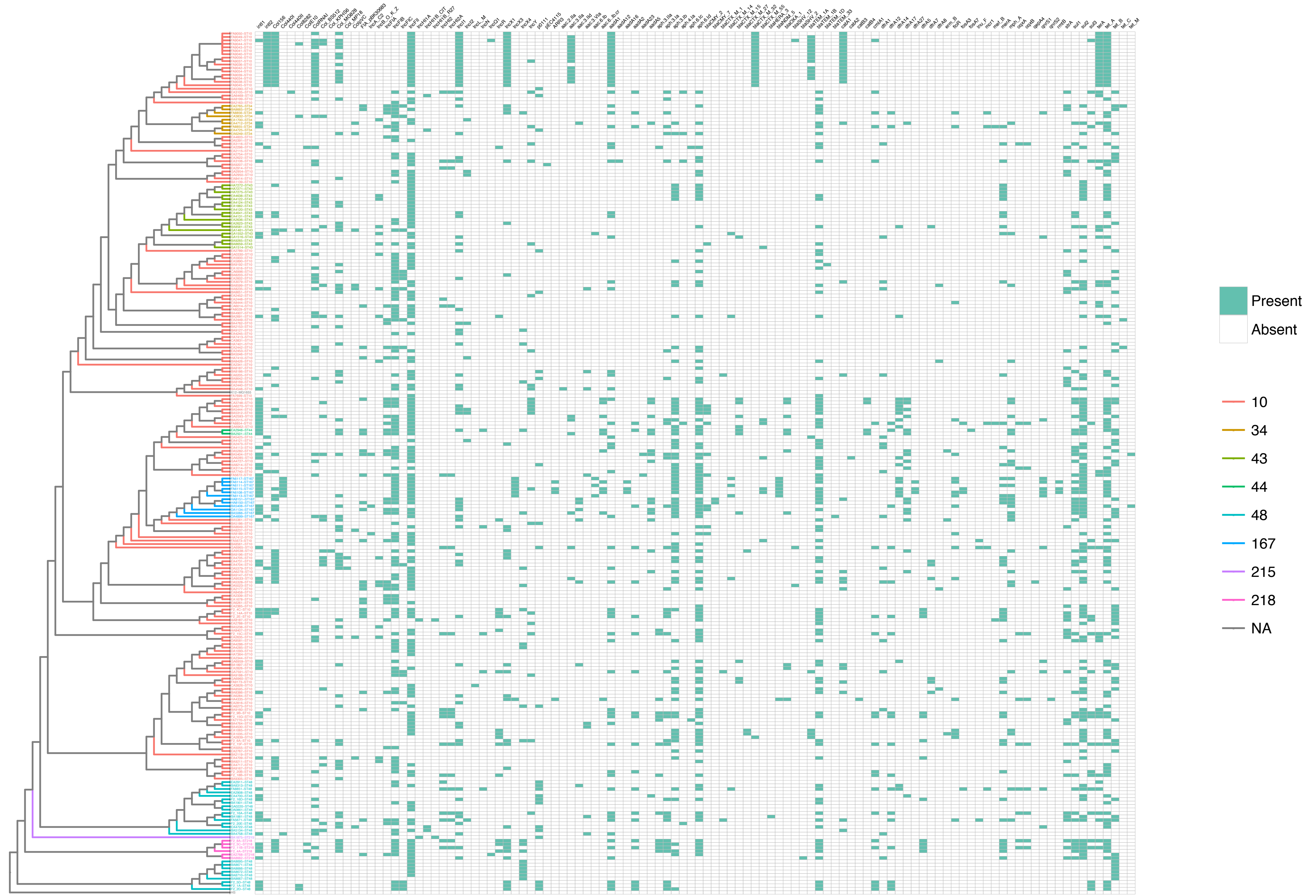

Figure S3. Heatmap depicting maximum-likelihood phylogeny from recombination filtered full core alignment (shown in figure 1) mapped against plasmid and antimicrobial resistance gene presence and absence. Tips are coloured by Achtman sequence type.
